# Accelerated epigenetic aging as a risk factor for chronic obstructive pulmonary disease and decreased lung function in two prospective cohort studies

**DOI:** 10.18632/aging.103784

**Published:** 2020-08-03

**Authors:** Miyuki Breen, Jamaji C. Nwanaji-Enwerem, Stefan Karrasch, Claudia Flexeder, Holger Schulz, Melanie Waldenberger, Sonja Kunze, Markus Ollert, Stefan Weidinger, Elena Colicino, Xu Gao, Cuicui Wang, Jincheng Shen, Allan C. Just, Pantel Vokonas, David Sparrow, Lifang Hou, Joel D. Schwartz, Andrea A. Baccarelli, Annette Peters, Cavin K. Ward-Caviness

**Affiliations:** 1 Oak Ridge Institute for Science and Education (ORISE), Center for Public Health and Environmental Assessment, US Environmental Protection Agency, Chapel Hill, NC 27709, USA; 2 Department of Environmental Health, Harvard T.H. Chan School of Public Health, Boston, MA 02115, USA; 3 MD-PhD Program, Harvard Medical School, Boston, MA 02115, USA; 4 Belfer Center for Science and International Affairs, Harvard Kennedy School of Government, Cambridge, MA 02138, USA; 5 Institute and Outpatient Clinic for Occupational, Social and Environmental Medicine, Inner Clinic, University Hospital of Munich, Ludwig-Maximilians-Universität, Munich, Germany; 6 Institute of Epidemiology, Helmholtz Zentrum München, Neuherberg, Germany; 7 Comprehensive Pneumology Center Munich (CPC-M), Member of the German Center for Lung Research (DZL), Munich, Neuherberg, Germany; 8 Research Unit Molecular Epidemiology, Helmholtz Zentrum München, Neuherberg, Germany; 9 Department of Infection and Immunity, Luxembourg Institute of Health, Esch-sur-Alzette, Luxembourg; 10 Odense Research Center for Anaphylaxis, Department of Dermatology and Allergy Center, University of Southern Denmark, Odense, Denmark; 11 Department of Genetic Dermatology, University of Kiel, Kiel, Germany; 12 Department of Environmental Medicine and Public Health, Icahn School of Medicine at Mount Sinai, New York, NY 10029, USA; 13 Department of Environmental Health Sciences, Mailman School of Public Health, Columbia University, New York, NY 10032, USA; 14 Department of Population Health Sciences, University of Utah, School of Medicine, Salt Lake City, UT 84132, USA; 15 Veterans Affairs Normative Aging Study, Veterans Affairs Boston Healthcare System, Department of Medicine, Boston University School of Medicine, Boston, MA 02118, USA; 16 Department of Preventive Medicine, Feinberg School of Medicine, Northwestern University, Chicago, IL 60611, USA; 17 Center for Public Health and Environmental Assessment, US Environmental Protection Agency, Chapel Hill, NC 27709, USA

**Keywords:** DNA methylation age, COPD, lung function, accelerated aging

## Abstract

Chronic obstructive pulmonary disease (COPD) is a frequent diagnosis in older individuals and contributor to global morbidity and mortality. Given the link between lung disease and aging, we need to understand how molecular indicators of aging relate to lung function and disease. Using data from the population-based KORA (Cooperative Health Research in the Region of Augsburg) surveys, we associated baseline epigenetic (DNA methylation) age acceleration with incident COPD and lung function. Models were adjusted for age, sex, smoking, height, weight, and baseline lung disease as appropriate. Associations were replicated in the Normative Aging Study. Of 770 KORA participants, 131 developed incident COPD over 7 years. Baseline accelerated epigenetic aging was significantly associated with incident COPD. The change in age acceleration (follow-up – baseline) was more strongly associated with COPD than baseline aging alone. The association between the change in age acceleration between baseline and follow-up and incident COPD replicated in the Normative Aging Study. Associations with spirometric lung function parameters were weaker than those with COPD, but a meta-analysis of both cohorts provide suggestive evidence of associations. Accelerated epigenetic aging, both baseline measures and changes over time, may be a risk factor for COPD and reduced lung function.

## INTRODUCTION

Lung disease continues to be a primary determinant of morbidity and mortality worldwide. A study on the global burden of disease indicates that although the contribution of asthma to global mortality decreased by 11.7% between 2005 and 2015, asthma still contributed to > 397,000 deaths in 2015. Chronic obstructive pulmonary disease (COPD) is a substantially greater contributor to global mortality with 3,188,300 deaths linked to COPD in 2015, and, unlike asthma, COPD-attributable deaths appear to be increasing [1]. A globally aging population is likely a primary contributor to the increased mortality burden of COPD, a trend which may exacerbate as the population of older adults (particularly in developed nations) rapidly expands.

Though lung function and lung disease are closely linked with aging [2], relatively little is known about how molecular indicators of biological age relate to lung disease and lung function. One of the most widely used biomarkers of biological age is telomere length [3]. Telomeres are repetitive sequences that cap the ends of chromosomes and prevent the loss of genetic information during DNA replication. Decreased telomere length is an indicator of increased biological age, and is associated with pulmonary fibrosis and emphysema, suggesting a link between molecular biomarkers of aging and lung disease [4–7]. Recently, DNA methylation has emerged as a novel biomarker for the aging process [8]. DNA methylation-based aging biomarkers are associated with mortality, cancer, hemostasis, frailty, and other age-associated traits and diseases [9]. However, relatively little is known about how lung disease and function relate to DNA methylation-derived aging biomarkers. In a study of two adult cohorts, DNA methylation loci associated with changes in lung function were correlated with DNA methylation age and age acceleration difference (difference between DNA methylation age and chronological age) [10]. In a study of adult women, accelerated DNA methylation aging was associated with incident lung cancer [11] and there have been two smaller studies examining asthma in children [12] and lung function in adults aged 70+ [13] showing positive associations. However, there has still been no large, prospective cohort study of lung function and lung disease risks using epigenetic aging biomarkers. Here we use the Horvath DNA methylation age (DNAmAge) biomarker [14] to determine if accelerated epigenetic aging is related to lung function measurements and incident COPD in two prospective, population-based cohorts.

## RESULTS

The characteristics of KORA are shown in Table 1. The mean age at baseline was 47.4 yr and there was an average of 7.09 yr between baseline and follow-up examinations. We observed 131 cases of incident COPD in KORA. The mean age acceleration difference (AAD) at baseline was 1.59 yr while the mean extrinsic epigenetic age acceleration difference (EEAD) at baseline was 2.90 yr. At follow-up, the mean lung function measures were 4.25 liters for FVC, 3.30 liters for FEV_1_, 2.93 liters for FEF25-75 and 0.78 for FEV_1_/FVC. The characteristics of the replication NAS cohort are shown in Supplementary Table 1. We define AAD as the difference between Horvath epigenetic age and chronological age. EEAD is defined as the difference between extrinsic epigenetic age and chronological age.

**Table 1:** Clinical Covariates for KORA participants.

*N* = 770*	**Baseline**	**Follow-up**
	**Mean (SD)**	**Mean (SD)**
Age (yr)	47.4 (5.57)	54.5 (5.60)
Follow-up time (yr)	-	7.09 (0.42)
Weight (kg)	77.8 (15.7)	-
Height (cm)	169 (9.13)	-
DNAmAge (yr)	50.0 (6.62)	54.9 (5.74)
AAD (yr)	2.59 (4.64)	0.36 (4.43)
EEAD (yr)	1.90 (5.65)	8.87 (5.78)
FVC (liter)	-	4.25 (0.97)
FEV_1_ (liter)	-	3.30 (0.77)
FEF25-75 (liter/s)	-	2.93 (1.01)
FEV_1_/FVC	-	0.78 (0.06)
**Binary Variables**	**N (%)**	**N (%)**
Female	406 (52.7)	406 (52.7)
Incident COPD	-	131 (18.0)
Asthma	52 (6.92)	81 (10.5)
Never Smoker	295 (38.4)	294 (38.2)
Former Smoker	279 (36.3)	328 (42.4)
Current Smoker	195 (25.4)	148 (19.2)

AAD = age acceleration difference; BMI = body mass index; COPD = Chronic obstructive pulmonary disease; DNAmAge = DNA methylation age; EEAA = extrinsic epigenetic age acceleration; EEAD = extrinsic epigenetic age acceleration difference; FEV_1_ = forced expiratory volume in 1 s; FVC = forced vital capacity; FEF25-75 = forced expiratory flow at 25-75% of FVC; GOLD = Global Initiative for Chronic Obstructive Lung Disease; GLI = Global Lungs Initiative. * = only 726 participants had values available for lung function outcomes.

**Figure 1:**
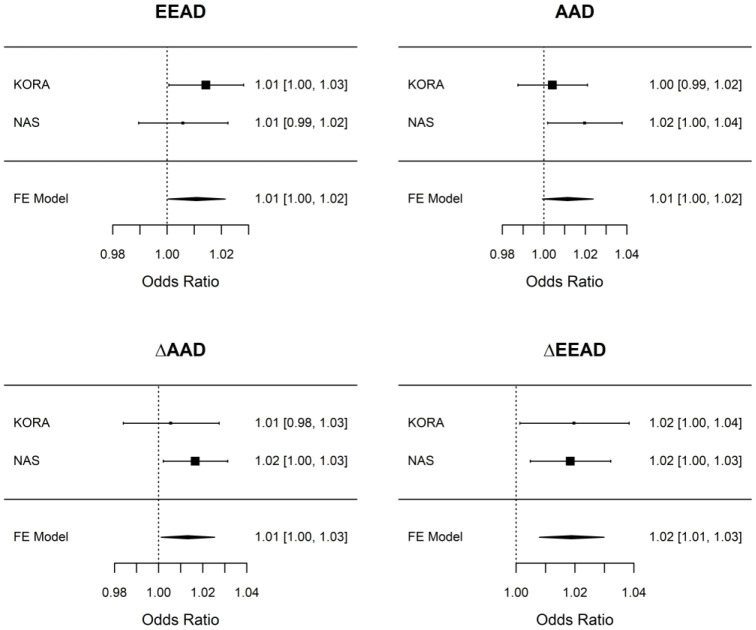
**Associations between epigenetic aging measures and COPD in KORA and NAS.** Associations were performed independently in KORA and NAS using the adjustment models laid out in Table 2 (including adjustment for baseline epigenetic age in the ΔAAD and ΔEEAD models) and are presented per 5-year change in the epigenetic aging measure. A fixed effect meta-analysis was used to combine results across cohorts. Only ΔEEAD is considered to have replicated as it had association with P < 0.05 in the discovery cohort (KORA) and in the replication cohort (NAS). For all epigenetic aging measures we consistently observe a 1-2% increase in the odds of COPD per 5-year change (elevation) in epigenetic aging. AAD = age acceleration difference; ΔAAD = change in age acceleration difference; EEAD = extrinsic epigenetic age acceleration difference; ΔEEAD = change in extrinsic epigenetic age acceleration difference; FE = Fixed effect.

Adjustment models for the analyses are given in Table 2 and described in the methods. Higher baseline EEAD was associated with higher odds of incident of COPD (odds ratio [OR] = 1.01 per 5-year higher EEAD; 95% confidence interval = 1.00 – 1.03) while AAD did not show an association with incident COPD (Table 3; Supplementary Table 2). There was a modest, negative correlation between the difference in AAD between baseline and follow-up (ΔAAD) and AAD as well as between the difference in EEAD between baseline and follow-up (ΔEEAD) and EEAD at baseline indicating that those with lower baseline AAD and EEAD had larger changes in these aging biomarkers over time (Supplementary Figure 1). Analyses of ΔAAD and ΔEEAD in KORA revealed stronger associations than baseline measures alone, and the overall pattern of associations for both ΔAAD and ΔEEAD was concordant with associations observed for AAD and EEAD, i.e. stronger associations for ΔEEAD than ΔAAD and a slight attenuation of the estimates with inclusion of additional confounders. ΔEEAD was associated with incident COPD (Table 3). Of the COPD associations with P < 0.05 in KORA, the ΔEEAD and COPD association replicated in NAS (Supplementary Table 3) with a nearly identical effect estimate and 95% confidence interval across the two studies and a smaller 95% confidence interval in the meta-analysis (OR = 1.02; 95% CI = 1.01-1.03; P = 7.1x10^-4^; Figure 1).

**Table 2:** Model descriptions.

**Outcome**	**Exposure (Epigenetic Aging Measure)**	
	**AAD, EEAD**	**ΔAAD, ΔEEAD**
Lung Function (FEV_1_, FVC, FEV_1_/FVC, FEF25-75)	age, sex, smoking status, height, weight, baseline COPD, baseline asthma	age, sex, smoking status, height, weight, baseline COPD, baseline asthma, years between examinations, baseline epigenetic age (AAD, EEAD)
COPD	age, sex, smoking status, height, weight	age, sex, smoking status, height, weight, years between examinations, baseline epigenetic age (AAD, EEAD)

List of the covariates adjusted for in each combination of outcomes and epigenetic aging measures (exposures) examined. Age, sex, smoking status, height, and weight were included in all models. Models for lung function variables additionally included baseline COPD and asthma. Models for the change in epigenetic age over time (ΔAAD, ΔEEAD) additionally adjusted for baseline epigenetic age (AAD or EEAD for ΔAAD and ΔEEAD models respectively) as well as the follow up time (years between examinations. AAD = age acceleration difference; COPD = chronic obstructive pulmonary disease; EEAD = extrinsic epigenetic age acceleration difference; FEF27-75 = forced expiratory flow at 25-75% of pulmonary volume; FEV_1_ = forced expiratory volume in 1 second; FVC = forced vital capacity.

**Table 3:** Associations between epigenetic aging measures and COPD.

**Epigenetic aging measure**	**OR**	**LCI**	**UCI**	**P**
AAD	1.00	0.99	1.02	0.63
EEAD	1.01	1.00	1.03	0.04
ΔAAD	1.01	0.98	1.03	0.62
ΔEEAD	1.02	1.00	1.04	0.04

Adjustments for the epigenetic aging models are shown in Table 2. Associations are presented per 5-year change in the epigenetic aging measure. AAD = Age Acceleration Difference; EEAD = Extrinsic Epigenetic Age Acceleration Difference; LCI = lower 95% confidence interval; OR = odds ratio; P = p-value; UCI = upper 95% confidence interval; ΔAAD = change in AAD between baseline and follow-up; ΔEEAD = change in EEAD between baseline and follow-up.

In KORA, we observed little to no evidence for associations between lung function measures (FVC, FEV_1_, FEF25-75, and FEV_1_/FVC) measured at baseline and epigenetic aging biomarkers measured at follow up (Supplementary Table 2). Unlike with COPD, lung function associations did not improve substantially when examining ΔAAD or ΔEEAD and many of the associations weakened (Supplementary Table 2). In NAS we also observed weak evidence for associations between lung function and aging measures, with the exception of FEV_1_/FVC which was associated with both ΔEEAD and ΔAAD (Supplementary Table 3). Though weak to null in KORA and NAS individually, associations between epigenetic aging and lung function were often consistent between the two cohorts, and in a meta-analysis, associations with FEV_1_ and FEV_1_/FVC and epigenetic aging measures were observed (Supplementary Table 4).

## DISCUSSION

We observed significant associations between epigenetic aging and COPD in analyses of independent, prospective cohorts. In KORA, a 5 yr difference in EEAD at baseline was associated with an increased risk of incident COPD, a though the magnitude of the associations was smaller than associations seen with other traits [9]. The change in EEAD between the baseline and follow-up examinations (ΔEEAD) was more strongly associated with COPD at follow-up than baseline EEAD alone. We observed an inverse correlation between baseline epigenetic aging and the change in epigenetic age over time, which may represent regression towards the mean. The association between ΔEEAD and COPD replicated in NAS, an older, all male cohort from the USA, and was has similar magnitude with a smaller 95% confidence interval in the meta-analysis than in either cohort alone. As the aging rate has been stated to be constant across adulthood [15], it is possible that ΔEEAD measured between baseline and follow-up is largely reflective of the aging rate at baseline in these adult populations. Future studies should examine baseline epigenetic aging rates to determine if they are risk factors for COPD, independent of baseline epigenetic age itself.

Associations for lung function in KORA were substantially weaker than associations for incident COPD and were often consistent with a null association. Only in a meta-analysis of KORA and NAS were associations between epigenetic aging and lung function observed for EEAD and FEV_1_ (-0.96% change in FEV_1_ from median per 5 year higher EEAD, 95% confidence interval = -1.80 – -0.11; Supplementary Table 4). Thus, while there is some evidence for an association between epigenetic aging and lung function, these associations appear weaker than those observed for COPD and may be best explored in meta-analyses or significantly larger cohorts.

Accelerated epigenetic aging has been associated with mortality and several health outcomes, and while the drivers of accelerated epigenetic aging have not been conclusively determined, a number of environmental exposures are associated with epigenetic aging [9]. EEAD was used here to measure accelerated epigenetic aging and calculated as the difference between extrinsic epigenetic age and chronological age. Unlike AAD, which is tissue agnostic, EEAD is valid only in blood and strongly correlated with blood immune cell counts and is considered a biomarker for epigenetic aging related to the immune system. COPD and lung function are associated with local or systemic inflammation and alterations regarding the immune system [16–18], which may account for why EEAD uniformly showed stronger associations with COPD and lung function than AAD.

There have been two other studies to date to examine lung outcomes and epigenetic aging. In the first study repeated measures of epigenetic age and a variety of physical fitness related measures, including FEV_1_, at ages 70, 73, and 76 in the Lothian Birth Cohort (a cohort recruited out of the Lothian area of Scotland in 1936). In this study, accelerated epigenetic age, equivalent to the AAD measure here, was associated with decreased FEV_1_ [13]. Extrinsic epigenetic age was not developed until after this study and thus was not tested. The second study examined a cohort of children as part of Project Viva, a mother-child pair cohort recruited from Boston, Massachusetts. In this cohort extrinsic epigenetic age acceleration measured at mid-childhood (mean age = 7.8 yr) was associated with asthma in cross-sectional associations, however extrinsic epigenetic age acceleration at birth was not associated with asthma at mid-childhood [12].

Our study has several advantages over these previous analyses. It is the first study to examine both COPD and lung function for multiple epigenetic aging measures in the same cohort. This enabled a direct comparison of the relationships revealing that associations for COPD were stronger than those for lung function measures in this cohort. Another strength of our study is the sample size. With more than 700 participants in KORA and more than 400 in NAS, our study is larger than both previous studies which had samples sizes < 1000. Additionally, KORA had a younger age distribution (mean age at baseline = 47.4 yr) as compared to either previous study, and thus may be more generalizable to an average adult population. Our study also benefits from replication across independent cohorts sampled from different countries and with different demographic distributions, e.g. age and sex. KORA and NAS also differed in the mean change in epigenetic age seen between baseline and follow-up (though all odds ratios are expressed per 5-year higher epigenetic age), another potential source of heterogeneity. Despite these differences, associations were often concordant between KORA and NAS, highlighting the consistency of the observed associations. A limitation of our study and the previous studies is the limited ethnic diversity. Both KORA and the Lothian birth cohort are single ethnicity cohorts (German and Scottish respectively) and while Project Viva is a multi-ethnic cohort, the majority of the participants are of Caucasian ancestry, as is also the case with NAS. This limits the ability of current studies to verify that associations generalize to multiple ethnicities. The repeated DNA methylation samplings in KORA allowed us to compute the change in DNA methylation over time and associate that with lung function and disease at follow-up. The Lothian Birth Cohort also had longitudinal DNA methylation samplings but was only able to examine longitudinal associations with FEV_1_, and did not compare baseline associations with changes in epigenetic aging over time. Our observation that the change in epigenetic age over time may be more strongly associated with COPD than baseline epigenetic age acceleration provides useful information on alterations in risk as epigenetic age acceleration changes.

### Potential mechanisms

Though we are still at the beginning of understanding the impacts of epigenetic aging, it is important to consider the potential mechanisms that might underlie associations between epigenetic aging biomarkers and disease to put these associations in a more complete context. There are several potential mechanisms linking epigenetic age with both COPD and lung function. Cellular senescence has been previously linked to the pathogenesis of lung disease. Telomere length (a molecular marker of aging and cellular senescence) is linked to emphysema, COPD, and pulmonary fibrosis [4, 5, 7]. Epigenetic aging presents a further potential biomarker of cellular aging that linked to risk of lung disease and possibly declines in lung function. It is also possible that epigenetic aging is a mediator of environmental exposures linked to lung disease and function. Elevated air pollution exposure is associated with higher DNA methylation age [9, 19, 20], and there have been a few studies suggesting that DNA methylation alterations may mediate the effects of air pollution exposures on lung function [21–23]. Thus, it is possible that one of the links between epigenetic aging and lung function / disease lies in epigenetic mediation of environmental exposures like air pollution.

## CONCLUSIONS

We used adult participants from two independent, longitudinal cohort studies to uncover prospective associations between COPD and lung function and epigenetic aging. The results expand on the literature suggesting that accelerated epigenetic aging is a risk factor for chronic, age-related diseases, even after controlling for chronological age. The results supplement work which establish epigenetic mechanisms as an important part of the pathophysiologic pathways underlying lung function decrements and incident COPD. Further work is needed to functionally validate the results, establish if modifications of epigenetic aging biomarkers can alter future disease risk, and expand associations to other ethnic groups.

## MATERIALS AND METHODS

### Study population

The discovery data for this study were taken from the fourth baseline survey of the Cooperation for Health Research in the Region of Augsburg (KORA S4) and their follow-up (KORA F4). These data consist of cross-sectional population-based surveys from registered German residents in Augsburg and its two adjacent counties (Southern Germany) [24]. The KORA S4 study was conducted between 1999 and 2001 with 4261 participants with ages from 25 to 74 yr. Of these participants, 3080 also participated in the 7 yr follow-up KORA F4 study between 2006 and 2008. Information on lifestyle and medical history questionnaire were gathered by trained medical staff during a standardized interview, as well as an extensive standardized medical examination including the collection of blood samples for later clinical chemistry and genomic analyses. The details of this cohort have been previously published [25–27]. The Bavarian Medical Association Ethics Committee approved these studies and all participants provided written informed consent.

The Normative Aging Study (NAS) was used to replicate associations from the KORA cohort. The NAS was established by the U.S. Department of Veterans Affairs and is an ongoing longitudinal cohort study of male volunteers within the Greater Boston area. In 1963, the NAS began enrolling participants free of any chronic medical conditions. The NAS is now a closed cohort, but study participants continually return for onsite, detailed medical examinations every 3-5 years. During these study visits, anthropomorphic measurements are performed, biological specimens are collected, and data on smoking status and additional risk factors that may impact health are documented. Participants provided written informed consent to the VA Institutional Review Board (IRB). The Harvard T.H. Chan School of Public Health and the VA IRBs granted human subjects’ approval. Eligibility for our study sample required complete data for the exposures, outcomes, and covariates determined *a priori* for the present analysis. Given the analysis structure, for inclusion, participants were also required to have at least two study visits.

### Epigenetic aging measures

DNA methylation was assessed in KORA S4 and F4 and NAS using the Illumina Infinium HumanMethylation 450k array, and DNAmAge was calculated using the online calculator provided by the laboratory of Dr. Steve Horvath [14]. Quality control for the methylation data and use of the online calculator for KORA was previously described in detail by Ward-Caviness CK et al. [19]. DNA methylation was assessed similarly in NAS as it was in KORA [28]. Briefly for both cohorts study staff measured DNA methylation on whole blood DNA collected at each study visit for the participants. After performing bisulfite conversion on the DNA (EZ-96 DNA Methylation Kit, Zymo Research, Orange, CA, USA), DNA methylation was quantified using the Illumina HumanMethylation450k platform (Infinium HD Methylation protocol, Illumina, San Diego, CA, USA). In NAS, to ensure a similar age distribution across chips/plates and minimize batch effects, a two-stage age-stratified algorithm was used to randomize samples across plates. Samples where >5% of probes had a beadcount < 3 or > 1% of probes had a detection P-value >0.05 were removed. The remaining samples were pre-processed with Illumina-type background correction without normalization and normalized with dye-bias and beta-mixture quantile normalization adjustments. In KORA samples with detection P-values > 0.05 for 1% of probes and probes with detection P-values > 0.05 for 1% of samples were removed. Methylation data was normalized using quantile normalization and beta-mixture quantile normalization. For both cohorts methylation beta values were calculated as the ratio of the intensity of the methylated signal over the total (methylated and unmethylated) probe intensity signal with a constant of 100 added to the denominator to prevent large values with low intensity probes, per standard procedures.

For both KORA and NAS the methylation beta values were used to estimate epigenetic aging measures. For our analysis, we focused on two epigenetic measures: Age Acceleration Difference (AAD) and Extrinsic Epigenetic Age Acceleration Difference (EEAD). AAD assesses differences between DNAmAge and chronological age and is tissue agnostic [14]. EEAD is a blood-specific measures of aging that is the difference between chronological age and Extrinsic Epigenetic Age which correlates with immune cell counts [11]. Both epigenetic aging measures assess “aging” as the difference between the epigenetic age measure (DNAmAge and Extrinsic Epigenetic Age for AAD and EEAD respectively) and chronological age.

### COPD and lung function

Incident COPD at follow up (KORA F4) was the primary outcomes with secondary analysis of lung function measures. COPD (including chronic bronchitis) was assessed via a questionnaire provided at baseline to the KORA participants, and all participants reporting COPD were removed from the incident COPD analyses. At follow-up a more robust assessment of COPD was possible thanks to the availability of spirometric indices. The spirometric indices available at follow up were forced expiratory volume in 1 s (FEV_1_), forced vital capacity (FVC), FEV_1_/FVC, and forced expiratory flow between 25-75% of FVC (FEF25-75). In KORA spirometry was performed in accordance with standard recommendations [29]. Briefly, spirometry was performed on sitting subjects wearing noseclips using a pneumotachograph-type spirometer (Masterscope PC; CareFusion, Höchberg, Germany) with a resistance of 0.05 kPa·l-1·s-1 at 10 l·s-1 and a volume accuracy of ±5 ml [30]. Spirometry was performed according to international standards [29, 31] and according to American Thoracic Society guidelines [32]. In NAS, spirometry was measured in standing participants with a nose clip using a 10-litre water-filled survey-recording spirometer and an Eagle II minicomputer (Warren E. Collins, Braintree, Massachusetts). Asthma and smoking status (current, former, and never) were assessed via self-report or the via the American Thoracic Society (ATS) questionnaire [33].

To identify all potential cases, incident COPD was assessed as a positive indication via spirometry, i.e. the definitions based on Global Initiative for Chronic Obstructive Lung Disease (GOLD) or the Global Lungs Initiative (GLI) methods, as well as self-report via questionnaire for chronic bronchitis. The GOLD and GLI methods are defined as FEV_1_/FVC < 0.70 [34] and FEV_1_/FVC z-score < -1.6445 (5% lower limit of normal) [35], respectively.

In NAS only lung function measures were available thus we identified incident COPD cases as a positive via either the GOLD or GLI methods.

### Covariates

Demographic, anthropometric, and lifestyle data were obtained during cohort enrollment and at follow-up. Smoking status was categorized as smokers (including occasional smokers), ex-smokers and never smokers via self-report. In KORA S4, physical examinations were conducted to obtain height and weight, and body mass index (BMI) was calculated as weight (kg) divided by height (m) squared. Similarly, in NAS demographic, anthropomorphic, and lifestyle data were collected at baseline and at follow up including smoking status, age, sex, height, and weight. Baseline asthma and COPD status were collected via self-report in KORA. COPD was assessed in NAS using the GOLD and GLI spirometry based methods, as there baseline questionnaire assessment of COPD was not available in NAS.

### Statistical analysis

We conducted all statistical analyses using the R statistical language (R Core Development Team, version 3.4.3) [36]. Logistic and linear regression models were implemented for binary and continuous outcome variables, respectively. We analyzed each epigenetic aging measure separately for associations with COPD and lung function and used a nominal significance threshold of *P* < 0.05 to determine significance given the limited number of outcomes examined and correlations between outcome measures.

We performed two statistical analyses. First, a prospective analysis was conducted using baseline (KORA S4) epigenetic aging measures and COPD as well as lung function assessed at follow-up (KORA F4). Second, we assessed if the change (Δ) in AAD and EEAD between baseline and follow-up was associated with COPD or lung function assessed at follow-up. This analysis included an adjustment for baseline epigenetic aging. For the analyses we adjusted for age, sex, smoking status, height, and weight. We additionally considered inclusion of body mass index (height [m]/weight [kg]^2^) however this did not modify associations after including height and weight, so we decided to use the more parsimonious model.

For the secondary analyses of lung function, we allowed participants with COPD at baseline to participate in the analyses to maximize sample size. To prevent confounding by baseline COPD status we added an adjustment for both baseline COPD as well as baseline asthma (assessed via self-report) to the models. For models with ΔAAD and ΔEEAD as predictors, we added the follow-up time to all models to observe if adjusting for variations in follow-up times altered associations, as well as adjustment for baseline epigenetic age. Table 2 details the models used for each analysis.

Associations with P < 0.05 in KORA were replicated in the Normative Aging Study (NAS). We chose a nominal p-value as the replication threshold because lung function and COPD are highly correlated (as COPD was in part defined by lung function). Thus, the tests were not independent and a Bonferroni multiple testing correction would be overly conservative. We additionally used the *metafor* package [37] in R to perform fixed-effects meta-analyses for associations across the two cohorts. Though we examined association in NAS and under the meta-analysis of NAS and KORA, formal replication was only considered for those associations with P < 0.05 in the discovery cohort (KORA) and with the same direction of association and P < 0.05 in the replication cohort (NAS) per accepted standards.

## Supplementary Material

Supplementary Figure 1

Supplementary Tables
