# Supplementary material for: Accelerated epigenetic aging as a risk factor for chronic obstructive pulmonary disease and decreased lung function in two prospective cohort studies
Source: Aging (Albany NY). 2020 Aug 3;12(16):16539–54. doi: 10.18632/aging.103784 (PMC7485704; doi:10.18632/aging.103784)
Supplement: Supplementary Figure 1 [file aging-12-103784-s002..pdf]

SUPPLEMENTARY FIGURE

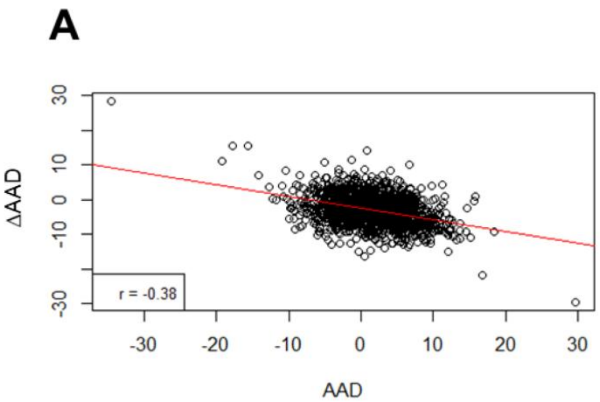

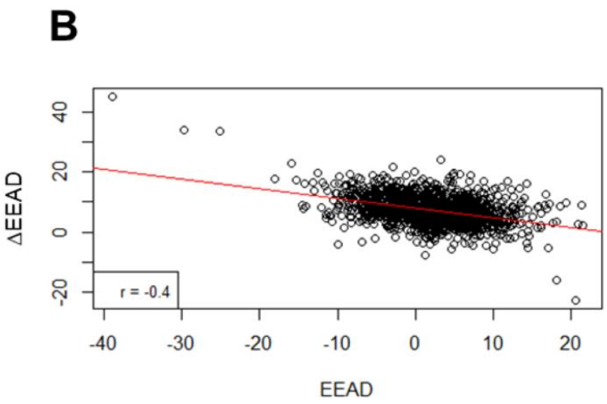

Supplementary Figure 1. Scatterplot and Pearson correlation coefficient. AAD and EEAD have a negative Pearson correlation (r) with ΔAAD (**A**) and ΔEEAD (**B**) respectively. AAD = age acceleration difference; EEAD = extrinsic epigenetic age acceleration difference; ΔAAD = change in age acceleration difference between baseline and follow-up examinations; ΔEEAD = change in extrinsic epigenetic age acceleration difference between baseline and follow-up.
