# Supplementary material for: Accelerated epigenetic aging as a risk factor for chronic obstructive pulmonary disease and decreased lung function in two prospective cohort studies
Source: Aging (Albany NY). 2020 Aug 3;12(16):16539–54. doi: 10.18632/aging.103784 (PMC7485704; doi:10.18632/aging.103784)
Supplement: Supplementary Tables [file aging-12-103784-s001..pdf]

## SUPPLEMENTARY TABLES

**Supplementary Table 1. Clinical covariates.**

| N = 408 | Baseline | Follow-up |
|---|---|---|
| | **Mean (SD)** | **Mean (SD)** |
| Age (yr) | 71.3 (6.36) | 77.6 (6.52) |
| Follow-up time (yr) | - | 6.26 (3.33) |
| Weight (kg) | 85.5 (14.1) | 83.4 (14.5) |
| Height (cm) | 173.9 (6.81) | 173.3 (7.06) |
| BMI (kg/m2) | 28.2 (4.03) | 27.7 (4.17) |
| DNAmAge (yr) | 72.1 (7.07) | 75.5 (7.00) |
| EEAA (yr) | 4.81(5.62) | 4.02 (7.78) |
| AAD (yr) | 0.77 (5.41) | -2.02 (7.72) |
| EEAD (yr) | 4.81 (5.62) | 4.02 (7.78) |
| FVC (liter) | 3.39 (0.66) | 3.37 (0.71) |
| FEV1 (liter) | 2.57 (0.58) | 2.48 (0.59) |
| FEF25-75 (liter/s) | 4.24 (1.83) | 3.54 (1.53) |
| FEV1/FVC | 0.76 (0.07) | 0.74 (0.07) |
| Binary Variables | N (%) | N (%) |
| Female | **0 (0)** | **0 (0)** |
| COPD GOLD (Incidence) | - | 0 (0) |
| COPD GLI (Incidence) | - | 15 (3.68) |
| White | 399 (97.8) | 399 (97.8) |
| Black | 5 (1.23) | 5 (1.23) |
| Hispanic | 4 (0.98) | 4 (0.98) |
| Ex-smoker | 260 (63.7) | 261 (64.0) |
| Current-smoker | 17 (4.17) | 17 (4.17) |

Clinical covariates for the NAS study participants. BMI = body mass index; DNAmAge = DNA methylation age; EEAA = extrinsic epigenetic age acceleration; IEAA = intrinsic epigenetic age acceleration; AAD = age acceleration difference; EEAD = extrinsic epigenetic age acceleration difference; FEV1 = forced expiratory volume in 1 s; FVC = forced vital capacity; FEF25-75 = forced expiratory flow at 25-75% of FVC; COPD = Chronic obstructive pulmonary disease; GOLD = Global Initiative for Chronic Obstructive Lung Disease; GLI = Global Lung Initiative.

**Supplementary Table 2. Associations between epigenetic aging biomarkers and COPD (a) and lung function (b) in KORA.**

| **a. COPD** | | | | | |
|---|---|---|---|---|---|
| | **Epigenetic aging measure** | **Odds ratio** | **LCI** | **UCI** | **P** |
| COPD | AAD | 1.00 | 0.99 | 1.02 | 0.63 |
| COPD | EEAD | 1.01 | 1.00 | 1.03 | 0.04 |
| COPD | ΔAAD | 1.01 | 0.98 | 1.03 | 0.62 |
| COPD | ΔEEAD | 1.02 | 1.00 | 1.04 | 0.04 |
| **b. Lung function** | | | | | |
| | **Epigenetic aging measure** | **Effect estimate** | **LCI** | **UCI** | **P** |
| FEV1 (% difference from median) | AAD | -0.43 | -1.64 | 0.77 | 0.48 |
| FVC (% difference from median) | AAD | -0.17 | -1.20 | 0.86 | 0.74 |
| FEV1/FCV (% difference from median) | AAD | -0.22 | -0.81 | 0.37 | 0.46 |
| FEF25-75 (% difference from median) | AAD | -1.61 | -4.05 | 0.83 | 0.20 |
| FEV1 (% difference from median) | EEAD | -0.91 | -1.89 | 0.07 | 0.07 |
| FVC (% difference from median) | EEAD | -0.62 | -1.45 | 0.22 | 0.15 |
| FEV1/FCV (% difference from median) | EEAD | -0.32 | -0.80 | 0.16 | 0.19 |
| FEF25-75 (% difference from median) | EEAD | -1.34 | -3.32 | 0.65 | 0.19 |
| FEV1 (% difference from median) | ΔAAD | -0.85 | -2.41 | 0.71 | 0.29 |
| FVC (% difference from median) | ΔAAD | -0.65 | -1.99 | 0.68 | 0.34 |
| FEV1/FCV (% difference from median) | ΔAAD | -0.10 | -0.86 | 0.66 | 0.80 |
| FEF25-75 (% difference from median) | ΔAAD | -0.85 | -3.98 | 2.27 | 0.59 |
| FEV1 (% difference from median) | ΔEEAD | -0.54 | -1.82 | 0.74 | 0.41 |
| FVC (% difference from median) | ΔEEAD | -0.10 | -1.19 | 0.99 | 0.86 |
| FEV1/FCV (% difference from median) | ΔEEAD | -0.54 | -1.16 | 0.08 | 0.09 |
| FEF25-75 (% difference from median) | ΔEEAD | -1.17 | -3.74 | 1.39 | 0.37 |

AAD = age acceleration difference; ΔAAD = change in age acceleration difference between baseline and follow-up exams; COPD = chronic obstructive pulmonary disease; EEAD = extrinsic epigenetic age acceleration difference; ΔEEAD = change in extrinsic epigenetic age acceleration difference between baseline and follow-up exams; FEF25-75 = forced expiratory flow at 25-75% of pulmonary volume; FEV1 = forced expiratory volume in the first second; FVC = forced vital capacity; LCI = lower 95% confidence interval; P = p-value; UCI = upper 95% confidence interval.

**Supplementary Table 3. Associations between epigenetic aging measures and COPD (a) and lung function (b) in NAS.**

| a. COPD | | | | | |
|---|---|---|---|---|---|
| | Epigenetic aging measure | Odds ratio | LCI | UCI | P |
| COPD | ΔEEAD | 1.02 | 1.00 | 1.03 | 0.008044 |
| COPD | ΔAAD | 1.02 | 1.00 | 1.03 | 0.02502 |
| COPD | AAD | 1.02 | 1.00 | 1.04 | 0.03089 |
| COPD | EEAD | 1.01 | 0.99 | 1.02 | 0.485577 |

| b. Lung function | | | | | |
|---|---|---|---|---|---|
| | Epigenetic aging measure | Effect estimate | LCI | UCI | P |
| FEF25-75 | ΔEEAD | -2.55 | -5.21 | 0.10 | 0.06042 |
| FEF25-75 | ΔAAD | -2.33 | -5.19 | 0.53 | 0.111603 |
| FEF25-75 | EEAD | -2.23 | -5.50 | 1.04 | 0.182474 |
| FEF25-75 | AAD | -0.65 | -4.20 | 2.89 | 0.717814 |
| FEV1 | ΔAAD | -1.32 | -2.79 | 0.15 | 0.07916 |
| FEV1 | EEAD | -1.10 | -2.75 | 0.56 | 0.194244 |
| FEV1 | ΔEEAD | -0.73 | -2.10 | 0.64 | 0.297151 |
| FEV1 | AAD | 0.28 | -1.51 | 2.07 | 0.761846 |
| FEV1/FVC | ΔEEAD | -0.79 | -1.38 | -0.20 | 0.008856 |
| FEV1/FVC | ΔAAD | -0.79 | -1.42 | -0.15 | 0.015798 |
| FEV1/FVC | EEAD | -0.59 | -1.31 | 0.13 | 0.108924 |
| FEV1/FVC | AAD | -0.49 | -1.27 | 0.30 | 0.223369 |
| FVC | AAD | 0.96 | -0.66 | 2.58 | 0.244423 |
| FVC | ΔAAD | -0.49 | -1.83 | 0.85 | 0.47577 |
| FVC | EEAD | -0.31 | -1.81 | 1.19 | 0.683633 |
| FVC | ΔEEAD | 0.10 | -1.15 | 1.35 | 0.877678 |

AAD = age acceleration difference; ΔAAD = change in age acceleration difference between baseline and follow-up exams; COPD = chronic obstructive pulmonary disease; EEAD = extrinsic epigenetic age acceleration difference; ΔEEAD = change in extrinsic epigenetic age acceleration difference between baseline and follow-up exams; FEF25-75 = forced expiratory flow at 25-75% of pulmonary volume; FEV1 = forced expiratory volume in the first second; FVC = forced vital capacity; LCI = lower 95% confidence interval; P = p-value; UCI = upper 95% confidence interval.

**Supplementary Table 4. Meta-analysis of KORA and NAS for COPD (a) and lung function (b) outcomes.**

| **a. COPD** | | | | | |
|---|---|---|---|---|---|
| **Outcome** | **Epigenetic aging measure** | **OR** | **OR LCI** | **OR UCI** | **P** |
| COPD | EEAD | 1.01 | 1.00 | 1.02 | 0.043004 |
| COPD | AAD | 1.01 | 1.00 | 1.02 | 0.06543 |
| COPD | ΔAAD | 1.01 | 1.00 | 1.03 | 0.031643 |
| COPD | ΔEEAD | 1.02 | 1.01 | 1.03 | 0.000706 |
| **b. Lung function** | | | | | |
| | **Epigenetic aging measure** | **Effect estimate** | **LCI** | **UCI** | **P** |
| FEV1 | AAD | -0.21 | -1.21 | 0.79 | 0.677415 |
| FEV1 | EEAD | -0.96 | -1.80 | -0.12 | 0.025818 |
| FEV1 | ΔAAD | -1.10 | -2.17 | -0.03 | 0.044241 |
| FEV1 | ΔEEAD | -0.63 | -1.56 | 0.31 | 0.1883 |
| FVC | AAD | 0.16 | -0.71 | 1.02 | 0.72534 |
| FVC | EEAD | -0.54 | -1.27 | 0.19 | 0.145254 |
| FVC | ΔAAD | -0.57 | -1.52 | 0.37 | 0.236804 |
| FVC | ΔEEAD | -0.01 | -0.84 | 0.81 | 0.973866 |
| FEV1/FVC | AAD | -0.32 | -0.79 | 0.15 | 0.185455 |
| FEV1/FVC | EEAD | -0.40 | -0.80 | -0.01 | 0.046866 |
| FEV1/FVC | ΔAAD | -0.50 | -0.99 | -0.02 | 0.04325 |
| FEV1/FVC | ΔEEAD | -0.67 | -1.10 | -0.25 | 0.001988 |
| FEF25-75 | AAD | -1.30 | -3.31 | 0.71 | 0.204109 |
| FEF25-75 | EEAD | -1.58 | -3.27 | 0.12 | 0.068706 |
| FEF25-75 | ΔAAD | -1.65 | -3.76 | 0.46 | 0.124265 |
| FEF25-75 | ΔEEAD | -1.84 | -3.68 | 0.01 | 0.050812 |

AAD = age acceleration difference; ΔAAD = change in age acceleration difference between baseline and follow-up exams; COPD = chronic obstructive pulmonary disease; EEAD = extrinsic epigenetic age acceleration difference; ΔEEAD = change in extrinsic epigenetic age acceleration difference between baseline and follow-up exams; FEF25-75 = forced expiratory flow at 25-75% of pulmonary volume; FEV1 = forced expiratory volume in the first second; FVC = forced vital capacity; LCI = lower 95% confidence interval; P = p-value; UCI = upper 95% confidence interval.
